# The Effect of a Program to Improve Adherence to the Mediterranean Diet on Cardiometabolic Parameters in 7034 Spanish Workers

**DOI:** 10.3390/nu16071082

**Published:** 2024-04-07

**Authors:** Ignacio Ramírez Gallegos, Marta Marina Arroyo, Ángel Arturo López-González, Maria Teófila Vicente-Herrero, Daniela Vallejos, Tomás Sastre-Alzamora, José Ignacio Ramírez-Manent

**Affiliations:** 1 Investigation Group ADEMA SALUD, University Institute for Research in Health Sciences (IUNICS), 07010 Palma, Balearic Islands, Spain; ignacioramirezgallegos@gmail.com (I.R.G.); marinaarroyomarta@gmail.com (M.M.A.); correoteo@gmail.com (M.T.V.-H.); d.vallejos@eua.edu.es (D.V.); tsastre04@sonrie.com (T.S.-A.); joseignacio.ramirez@ibsalut.es (J.I.R.-M.); 2 Faculty of Dentistry, University School ADEMA, 07010 Palma, Balearic Islands, Spain; 3 Institut d’Investigació Sanitària de les Illes Balears (IDISBA), Balearic Islands Health Research Institute Foundation, 07010 Palma, Balearic Islands, Spain; 4 Balearic Islands Health Service, 07010 Palma, Balearic Islands, Spain; 5 Faculty of Medicine, University of the Balearic Islands, 07010 Palma, Balearic Islands, Spain

**Keywords:** Mediterranean diet, mobile SMS, cardiometabolic index

## Abstract

Background: Cardiovascular and metabolic diseases include a large group of pathologies and constitute one of the most serious chronic health problems facing the 21st century, with high rates of morbidity and mortality worldwide. Unhealthy diets influence the development of these pathologies. The Mediterranean diet can be an important part in the treatment of these diseases. The objective of this study was to assess the effect of a program that aims to increase adherence to the Mediterranean diet on the improvement of different cardiometabolic risk parameters. Methods: A prospective intervention study was carried out on 7034 Spanish workers. Prior to the intervention, 22 cardiometabolic risk scales were evaluated. Participants in this study were informed both orally and in writing of the characteristics and benefits of the Mediterranean diet and were given the website of the Ministry of Health, Consumption and Social Welfare of Spain, which provides advice on nutrition. Adherence to the Mediterranean diet was reinforced by sending a monthly SMS to their mobile phones. After six months of follow-up, the 22 risk scales were re-evaluated to assess changes. Means and standard deviations were calculated using Student’s *t* test to analyse quantitative variables. Prevalence was calculated using the Chi-square test when the variables were qualitative. Results: All the cardiometabolic risk scales studied decreased after implementing a program to improve and enhance adherence to the Mediterranean diet. The number of losses in the sample was very low, standing at 4.31%. Conclusions: The Mediterranean diet is effective in reducing all cardiovascular risk scales evaluated. The mean values and prevalence of high values of the different cardiometabolic risk scales analysed led to lower values after the implementation of the program to increase adherence to the Mediterranean diet. We observed a significant positive difference in metabolic age in both sexes. We have obtained a significant improvement in the insulin resistance index, especially in the SPISE-IR index, data that we have not found in previous publications. Easy access to the Internet and new information and communication technologies facilitate adherence to a diet and can reduce the number of losses.

## 1. Introduction

Cardiovascular and metabolic diseases include a large group of pathologies, among which the most prevalent are high blood pressure, obesity, and type 2 diabetes [1,2]. These diseases constitute one of the most serious chronic health problems facing the 21st century and are one of the most significant causes of morbidity and mortality worldwide [3,4,5]. Further, nowadays, unhealthy diets, alcohol and tobacco consumption, and a lack of physical exercise are much more prevalent at younger ages, which produces an increase in metabolic diseases in a much younger population [6,7,8,9,10].

Alongside the serious complications associated with these pathogenic groups, we must add the important socioeconomic impact that they entail. From an epidemiological perspective, cardiometabolic diseases are the most important problem in our society in terms of frequency, resource consumption, and mortality, thereby constituting a substantial problem for health systems, health managers, government authorities, epidemiology, and preventive medicine [11,12]. Total costs increase with increasing disease burden and technological advances [13,14,15]. To calculate costs, expenses related to risk factor control, medication use, and hospitalization are taken into account [16,17,18].

It is estimated that the European Union spent EUR 282 billion in 2021 on care for cardiometabolic diseases, of which healthcare and long-term care constituted 55% (EUR 155 billion). This cost represents 11% of EU health spending. Losses in labour productivity were estimated at 17% (EUR 48 billion), while the costs of informal care reached 28%. Spending per person ranged from EUR 381 in Cyprus to EUR 903 in Germany [19]. These pathologies carry a high economic burden for society, but research and intervention on healthy lifestyles can contribute to the prevention of these diseases and their complications, thus improving the quality of life of the population and reducing the economic burden [20,21,22,23,24].

Research on cardiometabolic risk factors has found behaviours that can increase or decrease the likelihood of developing cardiovascular disease [25,26,27,28]. Cardiometabolic risk factors have been classified into major and emerging risk factors. The WHO considers smoking, a sedentary lifestyle, a diet low in fibre and rich in cholesterol and saturated fats, diabetes mellitus, dyslipidaemia, and high blood pressure the main, modifiable cardiometabolic risk factors [29]. Furthermore, high-sensitivity C-reactive protein [30,31,32,33], ApoB [34], uric acid [35,36], and homocysteine [37,38] are considered emerging factors.

These antecedents make it necessary to set the metabolic health of the population as an objective, with special intensity on the younger population. This preventive provision is essential to stop this increase in metabolic diseases that is perpetuated into the next generations [39,40,41,42].

The Mediterranean diet is characterized by a high content of fruits, vegetables, fish, legumes, nuts, and olive oil and is considered one of the healthiest as it helps prevent cardiovascular diseases [43,44,45,46]; hence, the objective of this study was to assess the effect of a program to increase adherence to the Mediterranean diet on the improvement of different cardiometabolic risk parameters.

## 2. Materials and Methods

### 2.1. Participants

A prospective intervention study was carried out in 7034 Spanish workers between the ages of 18 and 69, 4038 of whom were men and 2996 women. This study was carried out between January and June 2022.

The inclusion criteria were established as follows:

- Being between 18 and 69 years of age;
- Belonging to one of the companies included in this study;
- Agreeing to participate in this study;
- Presenting low adherence to the Mediterranean diet at the beginning of this study.

**Figure 1:**
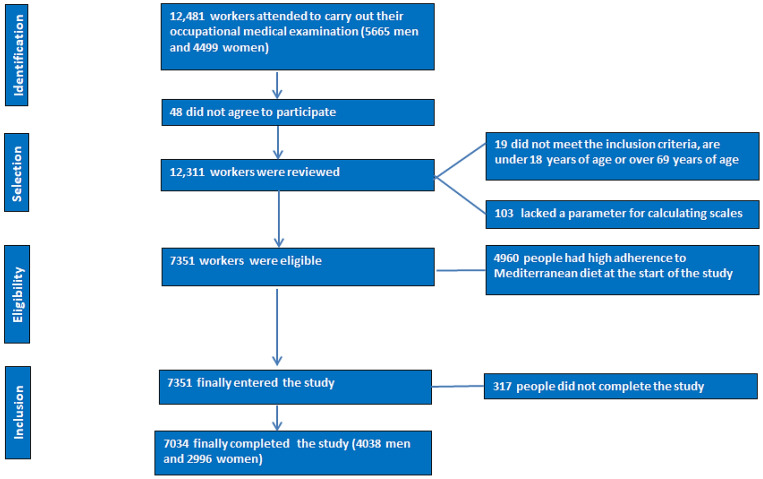
PRISMA flowchart of participants in this study.

A flowchart of the study participants is presented in Figure 1.

### 2.2. Determination of Variables

The health personnel of the occupational health services of the participating companies were responsible for obtaining the necessary data to carry out this study through the following:

- Anamnesis. A comprehensive clinical history in which data on sociodemographic variables such as age, sex, and adherence to the Mediterranean diet were collected.
- Anthropometric and clinical determinations. These included height, weight, waist and hip circumference, and systolic and diastolic blood pressure.
- Analytical determinations. The lipid profile and glycaemia were determined.

In an attempt to avoid possible biases in this study, the measurement techniques of the variables were standardized.

#### 2.2.1. Anthropometric Determinations

Height and weight were measured using a SECA 700 scale and a SECA 220 stadiometer. Measurements were taken with the person wearing underwear according to the international standards for anthropometric evaluation of the ISAK [47]. Data are expressed in centimetres and kilograms.

To assess the abdominal waist circumference, a SECA model measuring tape was used, placed mid-distance between the last rib and the iliac crest, and parallel to the floor. The person stood with their abdomen relaxed. The hip circumference was measured in the same position, placing the measuring tape also parallel to the floor at the level of the widest part of the buttocks [48,49].

Metabolic age, body fat percentage, and visceral fat percentage were obtained using a TANITA model MC-780 S MA bioimpedance meter.

Body mass index, waist/height ratio, and waist/hip ratio were calculated as overweight and obesity scales [50].

#### 2.2.2. Clinical Determinations

Blood pressure was determined with an OMRON-M3 model blood pressure monitor. For a correct assessment, the person was seated with their back resting on the back of the chair and had to rest for at least 10 min. The patient needed to be relaxed, with their arm supported and at heart level, without crossed legs. They should not have eaten, smoked, drunk alcohol or tea at least one hour before having their blood pressure taken. The cuff was placed around the arm on the skin, about 2–3 cm above the flexure of the elbow until it fitted well without being too tight. For this, cuffs are available in different sizes. Three consecutive determinations were carried out, at one-minute intervals. The figure obtained was an average of the three. 

#### 2.2.3. Analytical Determinations

Blood values were obtained by venepuncture after a prior 12 h fast. Then, samples were processed and refrigerated for adequate conservation for a time never exceeding 48–72 h. The analyses of the samples were carried out in reference laboratories that use a similar methodology. Triglycerides, total cholesterol, and blood glucose were determined using enzymatic techniques, while precipitation techniques were used for HDL cholesterol [51,52]. LDL cholesterol was estimated indirectly by applying the following Friedewald formula, which is valid provided that triglycerides do not exceed 400 mg/dL: LDL-c = total cholesterol -HDL-c- triglycerides/5. If the figure was greater than 400 mg/dL, the LDL was determined directly. All analytical variables are expressed in mg/dL.

#### 2.2.4. Risk Scales

The risk of insulin resistance was assessed using three scales, TyG index [53], METS-IR [54], and SPISE-IR [55], while the risk of non-alcoholic fatty liver was assessed with the lipid accumulation product scale [56]. For cardiovascular risk, the Framingham scale [57] and metabolic age were used [58]. Different risk scales for atherosclerosis, such as atherogenic dyslipidaemia [59], lipid triad [59], and atherogenic indices (cholesterol/HDL, LDL/HDL, and triglycerides/HDL) [60], were also determined. The presence of metabolic syndrome was also assessed by applying the NCEP ATP III and IDF criteria [61]. The formulas and cut-off points for these scales are presented in Table 1.

Adherence to the Mediterranean diet was determined by applying the PREDIMED questionnaire [62], which consists of 14 questions valued with 0 or 1 point. Values of nine points or more indicate a high adherence to the diet.

Once the variables had been evaluated, the people included in this study received both oral and written information about the characteristics and benefits of the Mediterranean diet. The recommendations to follow a Mediterranean diet are incorporated in the computer programs of the occupational health services of the autonomous communities that participated in this study. Hence, it was easy to print information for patients about recommendations for following this diet. As a complement to these tips, they were given the link to the website of the Ministry of Health, Consumption and Social Welfare of Spain [63], https://estilosdevidasaludable.sanidad.gob.es/ “URL (accessed on 7 February 2024)”, which provides advice on physical exercise, healthy lifestyle habits, how to eat healthily and plan menus, how to shop and cook healthy food, and false myths about food. As a reinforcement element, after receiving the permission of the study participants, a monthly SMS was sent to their mobile phone reminding them of the benefits of the Mediterranean diet and reinforcing their participation in this study. Multiple studies have demonstrated the usefulness of new technologies to enhance weight loss programs and modify lifestyles [64,65,66,67]. 

### 2.3. Statistical Analysis

Means and standard deviations were calculated using Student’s *t* test to analyse quantitative variables. Prevalence was calculated using the Chi-square test when the variables were qualitative. The SPSS 29.0 program was used to perform the statistical analysis. An accepted level of statistical significance was established as *p* = 0.05.

## 3. Results

There were 7034 participants in our study, with an average age at the beginning of this study of 47.1 years for men and 45.4 for women. The sample was made up of 57.4% men and 42.6% women. The pre- and post-counselling anthropometric, clinical, and analytical parameters are presented in Table 2. The results obtained show a clear improvement in all the parameters evaluated after the program, with higher values in men.

The mean values of the different cardiometabolic risk scales by sex and an established comparison of before and after the program are presented in Table 3. The mean values improved in all cardiometabolic risk scales after implementing the program to increase adherence to the Mediterranean diet. In all cases, the pre- and post-program differences are statistically significant, with a more noticeable improvement in the case of Framingham relative risk, AI total cholesterol/HDL-c, and AI LDL-c/HDL-c, which offers better results in women.

Table 4 shows the prevalence of high values of the different cardiometabolic risk scales, where the same trend already mentioned for the average values can be seen; that is, prevalence is lower in the post-program period and statistically significant in all cases.

## 4. Discussion

This study was carried out with the collaboration of the occupational health services of three autonomous communities (Balearic Islands, Madrid, and Valencian Community), with an average age of 47.1 years in men and 45.4 in women, which would correspond, approximately, to half of the life expectancy of the Spanish population [68]. Although, as mentioned above, our sample ranges between 18 and 69 years of age, we consider that the average age of the participants is important since the majority of them are between 40 and 50 years of age and, although the prevention of cardiometabolic risk is essential at any age and from childhood itself [69,70,71,72], our results demonstrate that modifying eating habits to a healthy diet improves the different cardiometabolic risk scales evaluated. There are multiple publications that refer to the modification of lifestyle habits, especially a healthy diet, as effective measures in the prevention of cardiometabolic risk factors [73,74,75,76,77]. 

In our study, we found that the values of all the cardiometabolic risk scales studied decreased after implementing a program to improve and enhance adherence to the Mediterranean diet. Some of these factors have been widely studied, and multiple publications are available on the benefits of the Mediterranean diet and the decrease in these risk factors once established, as is the case of high blood pressure [78,79,80], dyslipidaemia [81,82], hyperglycaemia [83,84], obesity [85,86], and the metabolic syndrome [87].

However, we have hardly found any studies that relate the improvement of other cardiometabolic risk scales that we evaluated in our work with diet. In reference to the Framingham scale, our results show a significant decrease in the risk calculated by this scale when following the Mediterranean diet. This association had already been published by Rubín-García et al. in reference to the PREDIMED-Plus study; however, the authors themselves recognized in their publication that since it was a cross-sectional study, it required other longitudinal studies to reinforce the cause–effect relationship [88]. The results of our study, with the pre- and post-monitoring results of the Mediterranean diet, contribute to demonstrating this association. In the case of metabolic age, it constitutes a marker of physical fitness and state of metabolic health that better predicts cardiovascular risk than chronological age. Under physiological conditions, the basal metabolic rate of an adult decreases between 1% and 2% for every 10 years of age. This loss is mainly due to sarcopenia, which is responsible for the decrease in energy consumption in adults [89]; however, a healthy lifestyle is capable of reducing this loss in basal metabolic rate. Thus, some studies determine that a difference of at least 12 years between chronological age and metabolic age reduces cardiovascular risk [90], while an increase in metabolic age in relation to chronological age raises said risk. In our work, we observed a significant positive difference in metabolic age, in both men and women, after following the Mediterranean diet. In the bibliographic review that we carried out, we found no other studies that compare the variation in metabolic age after the implementation of an adherence program to the Mediterranean diet.

Regarding the evaluation of insulin resistance, this was carried out using three formulas that have been widely recommended and accepted: TyG index, METS-IR, and SPISE-IR. In all of these, a significant reduction was obtained after the diet, finding the highest difference, for both men and women, with the METS-IR. Different previous studies found an improvement in the TyG index with diet [91,92], as shown in our analysis. In a previous cross-sectional study conducted by our research group, we found an association between people following a Mediterranean diet and a lower index of insulin resistance measured with METS-IR [54]. On this occasion, we carried out a longitudinal study in which we evaluated this pre- and post-intervention index (adherence to the Mediterranean diet), revealing a significant improvement in this index, and reinforcing the idea of the benefits afforded by the Mediterranean diet. Regarding the SPISE-IR index, we were unable to find any previous publication that associated its reduction with diet. 

The risk of non-alcoholic fatty liver disease was assessed using the lipid accumulation product (LAP) scale, in which we found a significant decrease in the mean between the values before the intervention and post-intervention. In this variable, it was not possible for us to evaluate the differences in the prevalence of high values of the scale pre- and post-intervention as we found no publications that establish the cut-off point. Neither did our bibliographic search locate any publication that evaluated the effect of the Mediterranean diet on the value of the LAP index. However, we did find a study conducted on patients with diabetes mellitus 2 and metabolic syndrome, which assessed the effects of increasing calcium consumption through the intake of skimmed milk together with a low-calorie diet, in which, among other variables, the index LAP was evaluated [93]. This study obtained a greater reduction in the LAP index when following a diet with high calcium content, which suggests that adherence to a healthier diet is capable of improving the LAP index, which may support our results. Other studies obtained an association between diet and the LAP index, but all of them were cross-sectional studies [94,95,96] and did not assess the changes after following a specific diet.

Regarding the atherogenic risk indices (total cholesterol/HDL-c, LDL-c/HDL-c, triglycerides/HDL-c), we also obtained a significant decrease in the high values of these indices after six months following the Mediterranean diet. Although the PREDIMED study showed the benefits of the Mediterranean diet on cardiovascular risk [97,98,99] and lipid profile [100,101], it did not carry out an assessment of the atherogenic risk indices that we evaluated. Other authors have also published a reduction in these indices with other types of diets [102,103], which may contribute to giving consistency to our results. 

In both atherogenic dyslipidaemia and the lipid triad, cardiometabolic risk decreased significantly after the intervention, with a greater reduction in the group of women for both indices. These results confirm those obtained in previous studies [104,105]. 

Finally, we also evaluated the effect of the Mediterranean diet on the metabolic syndrome (MetS). There are different formulas to evaluate the metabolic syndrome, However, they all have in common abdominal obesity, hypertriglyceridaemia, low levels of HDL-c cholesterol, high blood pressure, and insulin resistance. In our study, we used the criteria of the NCEP ATPIII (National Cholesterol Education Program Adult Treatment Panel) and the IDF (International Diabetes Federation). Although it is well known that the modification of dietary habits is the most important therapeutic component of MetS, the most effective diet in its treatment has not yet been established [106,107,108,109,110,111]. For this reason, we also wanted to include the results obtained with the Mediterranean diet in this cardiometabolic risk factor, finding a very high risk reduction with the two formulas used in both men and women.

As other authors have commented, the beneficial effects of a healthy diet are due to established dietary modifications rather than limiting the intake of certain nutrients. The Mediterranean diet allows for a series of dietary recommendations that are easy to understand and follow by patients, with a quality and variety of foods that are effective for the prevention of cardiometabolic diseases [112]. It also constitutes a type of sustainable diet, as it has a low environmental impact compared to other models of diet that involve greater nitrogen production and water consumption [113]. The benefits it brings for both human health and a sustainable diet with the lowest environmental impact has led the EAT-Lancet Commission to classify it as a healthy and sustainable diet recommended for the world’s population [114]. Our study corroborates the ease of following the recommendations of a Mediterranean diet, also supported by the advice on the website of the Ministry of Health, Consumption and Social Welfare of Spain [63] and an SMS sent monthly reminding them of the benefits of the Mediterranean diet, as other papers have shown that adherence to diet and changes in lifestyle [115,116] can be reinforced through new information and communication technologies. In our work, 7351 people began the intervention with losses during its development of 317 people. Ultimately, this study had a completion rate of 95.69% of the initial sample compared to 4.31% losses, which we consider very low, since, in the published literature, losses range around 20% [117,118,119].

## 5. Strengths and Limitations

As a limitation of this study, it is worth highlighting that some variables, such as insulin resistance, non-alcoholic fatty liver, and atherogenic risk, among others, do not determine the pathological process but rather entail an increased risk of presenting it using risk scales.

Strengths include a large sample size (over 7000 people) and the great number of cardiometabolic risk scales analysed.

## 6. Conclusions

The Mediterranean diet has been shown to be effective in reducing all cardiovascular risk scales evaluated in our study.

The mean values and prevalence of high values of the different cardiometabolic risk scales analysed in this study, show lower values, after the implementation of the program to increase adherence to the Mediterranean diet. It is important to highlight a decrease in metabolic age in both sexes, which is associated with a decrease in cardiovascular risk. An improvement in the insulin resistance index, especially with regard to the SPISE-IR index, of which we have not found any previous publication that associates its reduction with diet. This constitutes a new contribution to scientific knowledge. Likewise, we have obtained an improvement in non-alcoholic fatty liver assessed using the LAP scale, and a decrease in atherogenic risk indices.

Monitoring and adherence to the diet has been reinforced and facilitated by new information and communication technologies and Internet access. This reduces the number of losses and may be useful in future studies. 

Our study was carried out in a working population between 18 and 69 years of age. However, as we have reported in our article, more and more younger people have health problems due to an inadequate diet. Based on the results obtained, we believe that the use of new technologies could be of great support in modifying the eating habits of younger people. We believe that this could open new research on the use of new technologies in adolescents to modify unhealthy lifestyle habits.

## Figures and Tables

**Table 1:** Formulas and cut-offs for the different cardiometabolic risk scales.

	Formula	Cut-Off
BMI	Weight/Height^2^	>30 kg/m^2^ obesity
WtHR	Waist/Height	>0.50
WtHipR	Waist/Hip	0.8 women; 0.95 men
TyG index	LN (triglycerides × glycaemia/2)	>8.5
METS-IR	LN (2 × glycaemia + triglycerides) × BMI/LN(HDL-c)	>50
SPISE	(=600 × HDL^0.185^/triglycerides^0.2^ × BMI^1.338^)	6.14
LAP	(waist (cm)-65) × triglyc (mMol) men; (waist (cm)-58) × triglyc (mMol) women	no cut-off
Atherogenic dyslipidaemia	high triglycerides + low HDL-c	
Lipid triad	Atherogenic dyslipidaemia + high LDL-c	
AI total cholesterol/HDL-c	Total cholesterol/HDL-c	>7 women; >9 men
AI LDL-c/HDL-c	LDL-c/HDL-c	>3
AI triglycerides/HDL-c	Triglycerides/HDL-c	>3

BMI: Body mass index. WtHR: Waist-to-height ratio. WtHipR: Waist-to-hip ratio. TyG index: Triglyceride glucose index. METS-IR: Metabolic score for insulin resistance. SPISE: Single-point insulin sensitivity estimator. LAP: Lipid accumulation product. HDL-c: High-density lipoprotein cholesterol. LDL-c: Low-density lipoprotein cholesterol.

**Table 2:** Characteristics of the population.

	Men Pre (*n* = 4038)	Men Post (*n* = 4038)		Women Pre (*n* = 2996)	Women Post (*n* = 2996)	
	Mean (SD)	Mean (SD)	*p*-Value	Mean (SD)	Mean (SD)	*p*-Value
Age	47.1 (8.0)	47.6 (8.1)	<0.001	45.4 (8.3)	46.0 (8.3)	<0.001
Weight	87.1 (13.1)	84.4 (13.0)	<0.001	78.2 (14.1)	72.2 (12.2)	<0.001
Waist	100.8 (9.1)	98.3 (9.6)	<0.001	104.3 (9.8)	98.7 (11.1)	<0.001
Hip	109.6 (9.4)	107.2 (9.7)	<0.001	114.8 (9.6)	110.8 (10.3)	<0.001
SBP	138.2 (19.0)	136.5 (18.8)	<0.001	130.2 (18.3)	127.3 (18.0)	<0.001
DBP	83.5 (11.7)	82.5 (11.8)	<0.001	80.1 (11.4)	78.1 (11.1)	<0.001
Total cholesterol	206.4 (38.9)	205.0 (39.4)	<0.001	204.8 (36.5)	200.7 (35.6)	<0.001
HDL-c	49.3 (10.9)	50.2 (10.8)	<0.001	57.5 (12.1)	58.6 (12.3)	<0.001
LDL-c	128.4 (33.6)	126.7 (34.1)	<0.001	126.1 (32.4)	122.3 (31.4)	<0.001
Triglycerides	144.0 (95.7)	138.6 (90.9)	<0.001	105.7 (57.7)	99.3 (57.9)	<0.001
Glycaemia	97.1 (24.3)	95.7 (23.4)	<0.001	93.2 (22.6)	91.6 (19.5)	<0.001

SBP: Systolic blood pressure. DBP: Diastolic blood pressure. HDL-c: High-density lipoprotein cholesterol. LDL-c: Low-density lipoprotein cholesterol. SD: Standard deviation.

**Table 3:** Mean values of different cardiometabolic risk scales according to sex and group.

	Men Pre (*n* = 4038)	Men Post (*n* = 4038)			Women Pre (*n* = 2996)	Women Post (*n* = 2996)		
	Mean (SD)	Mean (SD)	Difference	*p*-Value	Mean (SD)	Mean (SD)	Difference	*p*-Value
Metabolic age	14.4 (1.1)	13.9 (1.7)	−3.9	<0.001	12.9 (1.6)	11.7 (2.1)	−9.0	<0.001
BMI	28.9 (3.8)	28.1 (3.8)	−2.8	<0.001	29.8 (4.9)	27.7 (4.4)	−7.0	<0.001
WtHR	0.58 (0.05)	0.57 (0.06)	−1.7	<0.001	0.64 (0.07)	0.61 (0.07)	−4.7	<0.001
WtHipR	0.92 (0.06)	0.91 (0.06)	−1.1	0.017	0.91 (0.07)	0.89 (0.07)	−2.2	<0.001
% body fat	34.6 (5.7)	32.5 (6.5)	−6.1	<0.001	44.6 (4.2)	41.3 (5.1)	−7.4	<0.001
% visceral fat	16.6 (5.4)	15.0 (5.7)	−9.6	<0.001	11.1 (4.0)	9.6 (3.7)	−13.5	<0.001
ALLY heart age	9.0 (8.6)	8.5 (8.7)	−5.6	<0.001	6.5 (10.1)	4.9 (10.5)	−24.6	<0.001
Framingham relative risk	1.7 (0.9)	1.6 (0.9)	−5.9	0.028	1.4 (0.8)	1.3 (0.8)	−7.1	<0.001
TyG index	8.7 (0.6)	8.6 (0.6)	−1.1	<0.001	8.4 (0.5)	8.3 (0.5)	−1.2	<0.001
METS-IR	45.9 (8.2)	44.2 (8.2)	−3.7	<0.001	44.3 (9.2)	40.6 (8.2)	−8.4	<0.001
SPISE	5.4 (1.2)	5.7 (1.3)	5.6	<0.001	5.7 (1.4)	6.4 (1.5)	12.3	<0.001
LAP	58.9 (45.0)	53.4 (42.3)	−9.3	<0.001	49.3 (39.0)	46.1 (38.0)	−6.5	<0.001
AI total cholesterol/HDL-c	4.3 (1.1)	4.2 (1.0)	−2.3	0.031	3.7 (0.9)	3.5 (0.9)	−5.4	<0.001
AI LDL-c/HDL-c	2.7 (0.8)	2.6 (0.8)	−3.7	0.027	2.3 (0.7)	2.2 (0.7)	−4.3	<0.001
AI triglycerides/HDL-c	3.2 (2.5)	3.0 (2.4)	−6.3	<0.001	2.0 (1.5)	1.8 (1.5)	−10.0	<0.001
Metabolic syndrome nº factors	1.9 (1.2)	1.7 (1.2)	−10.5	<0.001	2.8 (1.0)	2.5 (1.1)	−10.7	<0.001

BMI: Body mass index. WtHR: Waist-to-height ratio. WtHipR: Waist-to-hip ratio. ALLY: Avoidable lost life years. TyG: Triglyceride glucose. METS-IR: Metabolic score for insulin resistance. SPISE: Single-point insulin sensitivity estimator. LAP: Lipid accumulation product. AI: Atherogenic index. HDL-c: High-density lipoprotein cholesterol. LDL-c: Low-density lipoprotein cholesterol.

**Table 4:** Prevalence of high values of different cardiometabolic risk scales according to sex and group.

	Men Pre (*n* = 4038)	Men Post (*n* = 4038)			Women Pre (*n* = 2996)	Women Post (*n* = 2996)		
	%	%	Difference	*p*-Value	%	%	Difference	*p*-Value
Metabolic age ≥ 12 years	68.2	53.3	−21.8	<0.001	16.02	9.8	−38.5	<0.001
Hypertension	47.9	43.5	−9.2	<0.001	30.7	24.6	−19.9	<0.001
High Total cholesterol	53.3	51.5	−3.4	<0.001	52.6	48.1	−8.6	<0.001
High LDL-c	46.5	44.5	−4.3	<0.001	42.7	37.6	−11.9	<0.001
High Triglycerides	34.3	31.5	−8.2	<0.001	16.2	13.2	−18.5	<0.001
Glycaemia > 125 mg/dL	7.8	7.3	−6.4	<0.001	5.0	3.6	−28.0	<0.001
BMI obesity	31.7	24.5	−22.7	<0.001	39.4	21.5	−45.4	<0.001
High WtHR	96.8	91.0	−6.0	<0.001	99.2	95.2	−4.0	<0.001
High WtHipR	96.8	92.3	−4.6	<0.001	95.9	91.7	−4.4	<0.001
Very high % body fat	100.0	87.3	−12.7	<0.001	100.0	82.9	−17.1	<0.001
High ALLY heart age	18.2	15.7	−13.7	<0.001	7.4	6.1	−17.6	<0.001
Framingham relative risk	30.9	28.7	−7.1	<0.001	9.6	8.9	−7.3	<0.001
High TyG index	39.1	35.7	−8.7	<0.001	24.2	19.6	−19.0	<0.001
High METS-IR	26.8	21.1	−21.3	<0.001	21.6	11.9	−44.9	<0.001
High SPISE	83.7	75.7	−9.6	<0.001	76.1	56.0	−26.4	<0.001
High AI total cholesterol/HDL-c	23.3	21.7	−6.9	<0.001	15.2	12.4	−18.4	<0.001
High AI LDL-c/HDL-c	32.7	31.6	−3.4	<0.001	16.3	12.7	−22.1	<0.001
High AI triglycerides/HDL-c	39.5	35.8	−9.4	<0.001	14.7	11.7	−20.4	<0.001
Atherogenic dyslipidaemia	10.1	8.3	−17.8	<0.001	12.7	9.7	−23.6	<0.001
Lipid triad	7.5	6.3	−16.0	<0.001	8.1	6.3	−22.2	<0.001
Metabolic syndrome NCEP ATPIII	27.5	22.5	−18.2	<0.001	23.7	20.5	−13.5	<0.001
Metabolic syndrome IDF	38.9	32.0	−17.7	<0.001	27.7	23.3	−15.9	<0.001

BMI: Body mass index. WtHR: Waist-to-height ratio. WtHipR: Waist-to-hip ratio. HDL-c: High-density lipoprotein cholesterol. LDL-c: Low-density lipoprotein cholesterol. ALLY: Avoidable lost life years. TyG: Triglyceride glucose. METS-IR: Metabolic score for insulin resistance. SPISE: Single-point insulin sensitivity estimator. LAP: Lipid accumulation product. AI: Atherogenic index. NCEP ATPII: National Cholesterol Education Program Adult Treatment Panel. IDF: International Diabetes Federation.

## Data Availability

This study’s data are stored in a database that complies with all security measures at the ADEMA-Escuela Universitaria. The Data Protection Delegate is Ángel Arturo López González.
